# Supplementary material for: Hybrid Models and Biological Model Reduction with PyDSTool
Source: PLoS Comput Biol. 2012 Aug 9;8(8):e1002628. doi: 10.1371/journal.pcbi.1002628 (PMC3415397; doi:10.1371/journal.pcbi.1002628)
Supplement: Text S4 — Complete source code for the PyDSTool package (version 0.88.120504). Includes API documentation and help files linking to web pages. This file is identical to the current public release on Sourceforge.net. (ZIP) [file pcbi.1002628.s004.zip › PyDSTool/html/PyDSTool.ModelConstructor'.GeneratorConstructor-class.html]

xml version="1.0" encoding="ascii"?


PyDSTool.ModelConstructor'.GeneratorConstructor


| Home | Trees | Indices | Help | PyDSTool |
| --- | --- | --- | --- | --- |

Package PyDSTool ::
Module ModelConstructor' ::
Class GeneratorConstructor

[hide private]

[frames] | no frames]

# Class GeneratorConstructor

source code

```
object --+
         |
        GeneratorConstructor
```

---


|  |  |
| --- | --- |
| Instance Methods | [hide private] |

|  |  |
| --- | --- |
| \_\_del\_\_(self) | source code |

|  |  |
| --- | --- |
| \_\_init\_\_(self, mspec=None, userevents=None, userfns=None, unravelInfo=True, inputs=None, checklevel=2, activateAllBounds=False, activatedBounds=None, targetGen=`'``'`, algparams=None, indepvar=`(``'``t``'``,` `[`-inf`,` inf`]``)`, tdata=None, parvalues=None, icvalues=None, reuseTerms=None, options=None, abseps=None, eventPars=None, preReuse=False, preReuseTerms=None, preFlat=False)  Notes for initialization arguments: | source code |

|  |  |
| --- | --- |
| activateBounds(self, varname=None, which\_bounds=`'``all``'`)  which\_bounds argument is either 'lo', 'hi', or a pair ('lo', 'hi'). | source code |

|  |  |
| --- | --- |
| addEvents(self, evtarg, eventPars=None) | source code |

|  |  |
| --- | --- |
| addFunctions(self, fnarg) | source code |

|  |  |
| --- | --- |
| getGenerator(self)  Build and return a Generator instance from an abstract specification. | source code |

|  |  |
| --- | --- |
| setForcedAuxVars(self, vlist) | source code |

|  |  |
| --- | --- |
| setOptions(self, optDict) | source code |

|  |  |
| --- | --- |
| setReuseTerms(self, rdict) | source code |

|  |  |
| --- | --- |
| setVfCodeInsertEnd(self, codestr) | source code |

|  |  |
| --- | --- |
| setVfCodeInsertStart(self, codestr) | source code |

**Inherited from `object`**:
`__delattr__`,
`__getattribute__`,
`__hash__`,
`__new__`,
`__reduce__`,
`__reduce_ex__`,
`__repr__`,
`__setattr__`,
`__str__`


|  |  |
| --- | --- |
| Properties | [hide private] |

**Inherited from `object`**:
`__class__`


|  |  |
| --- | --- |
| Method Details | [hide private] |

|  |  |
| --- | --- |
| \_\_init\_\_(self, mspec=None, userevents=None, userfns=None, unravelInfo=True, inputs=None, checklevel=2, activateAllBounds=False, activatedBounds=None, targetGen=`'``'`, algparams=None, indepvar=`(``'``t``'``,` `[`-inf`,` inf`]``)`, tdata=None, parvalues=None, icvalues=None, reuseTerms=None, options=None, abseps=None, eventPars=None, preReuse=False, preReuseTerms=None, preFlat=False)  *(Constructor)* | source code |

Notes for initialization arguments:

mspec : corresponding ModelSpec, for reference

userevents : list of Event objects userfns : dictionary of named user
functions specs inputs : dictionary of Variable objects algparams :
dictionary of algorithmic parameters for Generator parvalues : dictionary
of parameter values icvalues : dictionary of initial condition values
reuseterms : dictionary of reused terms in specifications

targetGen : STRING name of any compatible Generator class, e.g.
'Vode\_ODEsystem'

eventPars : list of parameter names associated solely with events

options : Internal use by ModelConstructor preReuse : Internal use
preFlat : Internal use

RETURNS: getGenerator method returns a Generator of the specified
class

Overrides: object.\_\_init\_\_

|  |  |
| --- | --- |
| activateBounds(self, varname=None, which\_bounds=`'``all``'`) | source code |

which\_bounds argument is either 'lo', 'hi', or a pair ('lo', 'hi').
Calling with no arguments activates all bounds.

  


| Home | Trees | Indices | Help | PyDSTool |
| --- | --- | --- | --- | --- |

|  |  |
| --- | --- |
| Generated by Epydoc 3.0.1 on Fri May 4 15:24:07 2012 | http://epydoc.sourceforge.net |
